# Transformational Leadership and Internal Communication as Predictors of Job Performance: A Perspective of Peruvian University Workers

**DOI:** 10.3390/bs16040588

**Published:** 2026-04-15

**Authors:** Inés Elena Jaimes-Soncco, Jéssica Karina Saavedra-Vásconez, Juan Luis Haro-Caceres, Edwin Octavio Cisneros-Gonzales, Dany Yudet Millones-Liza

**Affiliations:** 1 Unidad de Ciencias Empresariales, Escuela de Posgrado, Universidad Peruana Unión, Lima 15102, Peru; ijaimes@upeu.edu.pe (I.E.J.-S.); jessy@upeu.edu.pe (J.K.S.-V.); luis.haro@upeu.edu.pe (J.L.H.-C.); 2 Escuela Profesional de Contabilidad, Facultad de Ciencias Empresariales, Universidad Peruana Unión, Lima 15102, Peru; 3 Escuela Profesional de Administración, Facultad de Ciencias Empresariales, Universidad Peruana Unión, Lima 15102, Peru

**Keywords:** transformational leadership, internal communication, job performance

## Abstract

Transformational leadership and internal communication are widely studied variables in organizational management; however, their joint effect as simultaneous predictors of multidimensional job performance remains underexplored, particularly in Latin American higher education contexts. This study examines whether transformational leadership and internal communication jointly predict task performance, contextual performance, and counterproductive job performance among Peruvian university workers. A quantitative, non-experimental, cross-sectional, and predictive correlational design was applied to a sample of 385 workers from a private Peruvian university, analyzed through Partial Least Squares Structural Equation Modeling (PLS-SEM). Results confirm that both variables significantly predict job performance across its dimensions, with internal communication showing stronger predictive effects on task and contextual performance than transformational leadership. These findings contribute to organizational and management theory by proposing and validating a joint predictive model that addresses existing conceptual and empirical gaps in the literature, while providing evidence-based recommendations for leadership development and communication management in university institutions operating in emerging economy contexts.

## 1. Introduction

Scientific advances that explore knowledge, interaction, and organizational structure depend on the role of the leader, who also assumes the responsibility of promoting internal communication through the set of actions, procedures, and tasks aimed at achieving institutional objectives (Avolio et al., 2001; Phan, 2025; Seraj & Sundram, 2025), that is to say, it is precisely the leader who must direct his collaborators so that there is effective communication, even more so considering that they are the ones who face a large number of challenges to which each collaborator must adapt.

Moreover, communication is an act of transmitting or receiving information through a variety of internal and external means, methods, and techniques. It is the set of actions on which the progress of an organization depends, to a certain extent, since it allows efficient work to be carried out with successful collaboration, which allows functional work (Mikkola & Valo, 2020); despite the benefits of communication, organizations today face several challenges that disrupt proper work; therefore, there is a need for companies or organizations to implement effective and competent management methods that, apart from breaking down communication barriers, are also focused on reactivating commitment to the worker. To achieve this goal, organizations have been strategically adopting transformational leadership in recent years, as it has been proven that this actively promotes success towards achieving change objectives, leading to personal and organizational well-being by implementing strategic practices that improve job performance (Aldossari & Alanizan, 2025; Rojas Carrasco et al., 2020).

Addressing transformational leadership involves turning workers into creative people, motivated to achieve, committed, and identified with the organization, inspiring them to participate, think collectively, and work as a team in support of the company or organization’s mission and vision (Parra et al., 1997). Transformational leadership has a direct relationship and significant impact on workers’ job performance (Zhang et al., 2021). In this regard, Byantara et al. (2023) establish that the transformational leadership style is associated with the innovative behavior of workers, thus stating that the internal locus of control is essential to moderate the impact of transformational leadership on job performance; therefore, it is crucial to consider transformational leadership attitudes to cultivate innovative behavior in workers, thus generating a positive effect for organizations.

Now, when we refer to organizations, the literature on organizational behavior suggests that internal communication fulfills four main functions in an organization or company: control, motivation, emotional expression, and the provision of information (Robbins, 2004). This highlights that internal communication is crucial in influencing individual behavior. It also motivates workers by clarifying the work they perform, evaluating their performance, and offering guidelines to improve it, if necessary. This organizational control also plays a role in releasing emotions and fulfilling social needs, as its ultimate function is internal communication to facilitate the flow of information necessary for individuals to make informed decisions and evaluate options before taking action. In this regard, Bris (2000) states that internal communication reveals the level of interaction between people, as well as the degree of respect, quality, and speed with which messages are shared between members of an organization or company; in this context, an assessment of job performance is enhanced, which involves measuring and evaluating both professional conduct and the results or achievements attained (Balluerka et al., 2021; Phan, 2025).

Similarly, efficient internal communication fosters a sense of acceptance and commitment within the organization; in contrast, poor communication breeds disappointment and misunderstandings. Rezeki (2023) and Sousa et al. (2025) show how good communication can help an organization or company function smoothly and successfully. In contrast, the lack of internal communication can cause confusion and insecurity within the company, preventing workers from easily completing their tasks and thereby damaging their job performance.

Research on job performance indicates that it involves integrated systems aimed at improving each employee’s effectiveness, performance, and job actions. These systems are designed with consideration of the activities workers perform, the proposed goals and results they must achieve, their growth potential, and their contribution to the success of the organization or company (Bautista et al., 2020; Rodríguez-Marulanda & Lechuga-Cardozo, 2019). Thus, organizations or companies expect human resources management to take the initiative in leadership to achieve maximum employee performance and improve job performance significantly (Chiavenato, 2009).

Based on the previous paragraphs, it is important to consider the impact of transformational leadership and the role of internal communication in an organization. Therefore, a reward system must be considered for job performance, as good performance is the fundamental basis of an organization. Thus, Charry (2018) determines that one of the factors or strategic tools that impact good job performance both internally and externally is internal communication, so correctly transmitting the objectives and values of the organization or company to workers is a key factor in all environments, even more so considering that good job performance is essential to developing effectiveness and success so that the actions or behaviors of workers contribute to the fulfillment of organizational goals (Vásquez et al., 2021).

From another perspective, the academic sector (Fahmy et al., 2023) argues that transformational leadership has little direct impact on teachers’ job performance. According to this view, job satisfaction serves as a mediating factor between the two variables (Warni et al., 2021), providing a background that underscores its very important role in teachers’ performance and improvement. In this way, it is considered that when the worker is satisfied with his work, it becomes something pleasant, and he does not feel obliged to do it; under these circumstances, a management strategy that seeks to promote job performance among workers is the implementation of programs that focus on improving their performance, taking into account that an ideal complement for this purpose is worker satisfaction, without leaving aside the role assumed by the transformational leader who encourages them, thus creating a positive atmosphere that paves the way to good job performance (Briceño Acaro & Jiménez Jiménez, 2025).

It is known that job performance guided by a transformational leadership style through employee participation often benefits the organization by achieving a stable level of continuous job performance over time (Nazari et al., 2022); it is necessary to take into account the four main variables that can modify this behavior: the business organization, the training, motivation, and attitudes of the worker (Pineda et al., 2023). In this way, paying special attention to the worker’s actions and behaviors is important, as job performance is the most crucial asset an organization can have (Chiavenato, 2011).

Another way to achieve good job performance by employees is to measure the level of assertive communication that is maintained in organizations, since this also determines how the employee’s skills develop (Duque et al., 2017); in this sense (Luis Hernández Juárez et al., 2017) confirms that communication is a key factor in achieving adequate job performance by employees, being evidence of the development of their skills, the ability to solve problems, adaptability, and teamwork, which constitute an important factor that allows workers to successfully face challenges and perform more effectively in their work roles; by integrating these practices, organizations can strengthen their workforce and promote exceptional performance for mutual benefit (Pashanasi et al., 2021).

Although various studies in the scientific literature aim to measure job performance, they often combine factors such as job satisfaction, ideal working conditions, attitude towards work, organizational support, and commitment (Aguirre-Camarena et al., 2025; Hendri, 2025; Tarmeño-Bernuy et al., 2025). Scarcely any research addresses it jointly with transformational leadership and internal communication, treating it exclusively as a direct predictor. This fragmentation has limited understanding of job performance as a construct that can be influenced by factors such as leadership and internal communication simultaneously. At the conceptual level, previous research links both variables. It examines them separately or as mediators of other organizational outcomes, such as teacher retention (Liu et al., 2025), organizational advocacy (Dong et al., 2025), or job satisfaction (Kuczman et al., 2024), without proposing a joint model focused on job performance. Within the empirical plane, the findings provide evidence that internal communication has a stronger effect than transformational leadership on organizational outcomes (Kim & Meganck, 2025); others show that communication primarily serves as a moderating variable between leadership and employee performance (Belias et al., 2025). This avoids the consolidation of a clear explanatory model. This fragmentation limits the understanding of job performance as a construct simultaneously influenced by leadership and internal communication, a gap that is accentuated in the context of Peruvian university workers, who operate in complex organizational environments marked by academic, administrative, and management demands that require effective leadership and adequate communication channels to sustain optimal levels of performance. This context is highly important, given that the Peruvian university system has experienced significant regulatory expansion and intensification since University Law No. 30220 and the establishment of SUNEDU, generating organizational structures in which leadership management and internal communication are decisive for staff performance, for which empirical evidence remains scarce.

## 2. Literature Review

### 2.1. Definition of Terms

#### Transformational Leadership

Transformational leadership was originally conceptualized by (Burns, 1987) and then operationalized by (Bass et al., 1987), who refer to it as a leadership approach in which the leader awakens workers’ interests, fostering awareness, enabling them to participate in the fulfillment of the institution’s objectives and mission, and even putting the company’s interests before their own. In this context, transformational leaders are those who contribute by providing the necessary tools to workers to encourage them to meet the established goals, constituting within this fact the alignment of the interests of workers concerning the company (Budiadi, 2026; Salcedo, 2018; Varela & González, 2018); and according to (Akkaya, 2019) this leadership style goes beyond encouraging the achievement of goals; it inspires its followers, promoting in them an ideal way to overcome their limitations, thus achieving good job performance because it reduces the workload of its workers by allowing them to use other innovative and creative ways to develop their work (Xiong et al., 2023). Therefore, transformational leadership has important predictive power for job performance.

### 2.2. Job Performance

Job performance was initially conceptualized by Borman and Motowidlo (1993), who defined it as the set of behaviors that contribute to the fulfillment of organizational objectives. It is a characteristic that highlights the strengths of an organization, which come from its employees; in this way Castro Vázquez (2016) and Chênevert et al. (2013) claim that due to constant changes and the intention to remain in the market, good job performance brings positive and sustainable results; that is, business survival over time will depend on the capacity, competence, and knowledge of its employees. In this regard, Millones-Liza and García-Salirrosas (2022), Olinda and Mori (2020) and Smeets and Beach (2022) place special emphasis on job performance as an indicator of efficiency and effectiveness in achieving a specific objective, which is why it is worth establishing supervision policies for each collaborator. Additionally, job performance is defined as actions or behaviors reflected in the fulfillment of assigned tasks and the various responsibilities the position demands, and is also conceptualized as the competence each collaborator possesses and their contribution to the organization (Pérez, 2009; Princy et al., 2026). In this research, three dimensions have been addressed: task performance, contextual performance, and counterproductive performance. Task performance refers to the extent to which employees efficiently organize, prioritize, and complete their responsibilities. Regarding contextual performance, these are voluntary behaviors beyond the formal requirements of the role, such as taking initiative, facing challenges, and contributing to the organization. Moreover, counterproductive performance is behavior that harms the institution, either through negative perceptions or by creating problems in the workplace (Geraldo, 2022).

### 2.3. Internal Communication

It was defined by Welch and Jackson (2007) as the set of multidimensional communication processes between managers and their employees, a key element in the sharing of ideas among work teams. It is a tool that aims to convey a specific message, which requires managing it appropriately to organize the flow of data transferred from the organization to the workers (Brandolini et al., 2009; Dávalos-Mogollón et al., 2024). It is also considered the basis of organizational culture and a strategy that allows employers to connect with their employees, resulting in positive attitudes and behaviors that improve work results (Guerrero et al., 2022; López Damián & Yee Villanueva, 2024; Nadeak & Naibaho, 2020). In addition, internal communication has a design that promotes employee commitment to institutions, awakening a bond of belonging and understanding of institutional objectives (Achyuta et al., 2025; Kim & Uysal, 2025; Welch & Jackson, 2007). Due to its importance, specific guidelines are established for effective communication, which is based on the 7C: clarity (use of appropriate, exact and concrete words), concise (every message must have a main message, avoiding repetitive phrases), concrete (the message must be solid to avoid misinterpretations), correct (without grammatical errors and with correct spelling), coherent (the message must be logical and related to the main topic), complete (the text must contain the details it intends to state) and polite (it does not have aggressive or threatening tones; that is, it is friendly and honest) (Gomes et al., 2023; Miheso & Mukanzi, 2020; Aggarwal, 2016). This means that if internal communication is effective, coordination is facilitated, and teamwork is efficient and effective, which translates into correct job performance.

### 2.4. Hypothesis Development

#### 2.4.1. Transformational Leadership and Job Performance

There are various investigations regarding job performance. This study’s particularity is the link between this variable and transformational leadership. There is evidence that the role of this leadership style extends beyond the leader’s management to the influence he exerts on those around him and how they maximize job performance (Alloubani et al., 2019; Bhatti & Alyahya, 2021). In addition, some authors argue that the actions of transformational leaders influence employees’ behavior, so the incentives they provide are essential for good job performance (Aldossari & Alanizan, 2025; Buchholz & Eichenseer, 2019; Saleem et al., 2020; H. Shao et al., 2022).

Other research supporting this fact is described by (Aldossari & Alanizan, 2025; Chi et al., 2023; Clifton, 2019), who establish that since the transformational leader creates confidence in his workers, he also can encourage them and exercise motivational incentive practices, so his role is significant for the worker’s performance, allowing workers to perform their functions as they are supposed to and even carry out activities that go beyond their responsibilities as a sign of proactivity, avoiding making any mistakes each time. In this sense, it is highlighted that within the characteristics of the leader is the ability to generate satisfaction, loyalty, and respect, thus creating a work environment conducive to all workers establishing a collective goal that allows them to contribute to the organization, thus showing high job performance (Imhangbe et al., 2019; Madhu & Krishnan, 2005; Zulkarnen & Sudarme, 2017). Based on the above, transformational leaders are expected to improve task performance by setting clear goals and motivating proactive behavior (H1), foster contextual performance by encouraging voluntary contributions beyond formal roles (H2), and reduce counterproductive behaviors by building trust and a positive organizational climate (H3). Furthermore, the following hypotheses are established:

**H1.**  *Transformational leadership predicts job performance in tasks among Peruvian workers.*

**H2.**  *Transformational leadership predicts contextual job performance in Peruvian workers.*

**H3.**  *Transformational leadership predicts counterproductive job performance in Peruvian workers.*

#### 2.4.2. Internal Communication and Job Performance

Communication is a key factor that enables workers to share ideas, work as a team, and manage their workdays effectively. When communication is used assertively, workers’ abilities improve, enabling them to perform their tasks and assume their roles more efficiently (Ohunakin & Olugbade, 2022). Chan and Lai (2017) argue that communication optimizes teamwork, thereby positively impacting results. Additionally, Imam et al. (2023) establish that one way to involve workers in obtaining benefits from job performance is to increase communication between work teams and their bosses, and between bosses and their superiors.

In this context, it is highlighted that internal communication can be attractive and convincing when developed between peers (Carrico & Riemer, 2011; Gehrau et al., 2024; Singh & Denner, 2025) by providing factual, more credible information that workers can feel more comfortable transferring in their daily work activities. Based on the above, internal communication is expected to improve task performance by providing workers with the information and clarity necessary to fulfill their responsibilities (H4) efficiently, foster contextual performance by encouraging teamwork, initiative, and voluntary contributions beyond the formal requirements of the position (H5), and increase the reporting of counterproductive behaviors by creating open communication environments where workers feel empowered to express their work concerns openly (H6). The following hypotheses are proposed:

**H4.**  *Internal communication predicts job performance in tasks in Peruvian workers.*

**H5.**  *Internal communication predicts contextual job performance in Peruvian workers.*

**H6.**  *Internal communication predicts counterproductive job performance in Peruvian workers.*

**Figure 1:**
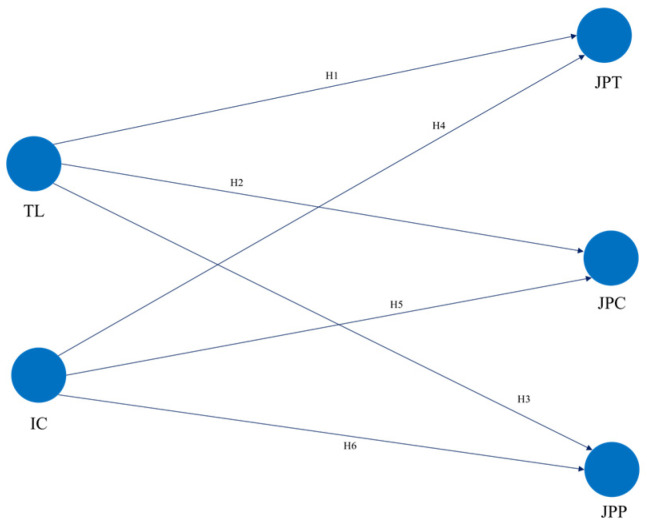
Structural model of transformational leadership and internal communication as predictors of job performance. Notes: TL: transformational leadership; IC: internal communication; JPT: job performance in tasks; JPC: context job performance; JPP: counterproductive job performance.

Based on the theories already mentioned in this research, the theoretical model based on the explored literature is presented below. See Figure 1.

## 3. Materials and Methods

This study aims to determine the impact of transformational leadership and internal communication on the job performance of Peruvian workers. For this purpose, the quantitative, non-experimental, cross-sectional, correlational, and predictive research method was used: quantitative because it worked with variables whose response scale is based on a numerical order; non-experimental because at no time during the study, the variables were manipulated; cross-sectional because the survey was taken at a single time, and correlational and predictive scope because it is intended to measure the relationship between the study variables, as well as establish these relationships to predict a future result based on a theoretical model (Ato et al., 2013; Bernal, 2010; Singh & Denner, 2025; Toro Jaramillo & Parra Ramírez, 2006). Regarding the surveys, authorization was initially requested from the ethics committee of the Universidad Peruana Unión. With this authorization, the survey was created on the Google Form platform, where informed consent was obtained. Each item was assessed on a 5-point Likert scale, ranging from 1 (never) to 5 (always).

Respecting the study instrument for transformative research, it is applied to the set of items proposed by van Beveren et al. (2017), named escalation, The Global Transformational Leadership (GTL), compiled by 07 items and published in a revised version indexed in Scopus with classification Q2, guaranteed as well. The instrument has been removed and validated in accordance with rigorous scientific standards, which are subject to revision by the user. For internal communication, the construct applied by (Papic Domínguez, 2019) consists of 11 items and was applied to the Chilean context, a context with Peruvian cultural proximity, selected for its cultural relevance for a Peruvian sample, thus following the principle of cultural equivalence, as Hambleton et al. (2005) point out that the cultural proximity between the instrument’s developmental context and the applied context is a determining factor in guaranteeing the validity of the inferences derived from the measurement. It should be noted that although items IC9 and IC10 have loadings below 0.7, they were retained in the analysis due to their high CR = 0.949 and adequate AVE = 0.630, which are considered good indicators (J. F. Hair et al., 2019). In addition, both capture substantive dimensions of internal communication according to the instrument of (Papic Domínguez, 2019) and their elimination could have compromised the construct’s content validity. Specifically, the two items capture the role of informal communication as a complement to and corrective of official communication, making it a theoretically relevant dimension in measuring the construct.

The scale of job performance applied to the Peruvian context (Geraldo, 2022) consists of 14 items. It is grouped into three dimensions: task performance (4 items), contextual performance (6 items), and counterproductive job performance (4 items). It is worth noting that, since the transformational leadership construct was in English, it underwent back-translation; for internal communication and leadership, the Spanish version was used, so no translation was required. Furthermore, all three instruments were administered to a focus group of six participants who met the study’s profile.

### 3.1. Sample and Procedure

The population consisted of workers from a private university across its three campuses, meeting the following inclusion criteria: full-time workers with a current contract who provide informed consent; and within the exclusion criteria: workers with a work suspension or senior management. In this instance, a non-probabilistic sampling method was applied at the researcher’s convenience for 2 months, yielding 385 respondents. The survey application was self-administered and shared through WhatsApp and official institutional channels (email). Table 1 shows the characteristics of the study population.

To address the possible common method variance (CMV) bias, procedural and statistical solutions were applied (Podsakoff et al., 2003). At the procedural level, participants’ anonymity was guaranteed. In addition, the items for the predictor variables were visually separated in the questionnaire, and the response scales were varied to reduce the effects of motive consistency. Statistically, the Harman single-factor test was performed by entering all items into an exploratory factor analysis; the first factor explained 38.23% of the total variance, well below the 50% threshold. This means that the common method variance does not introduce a significant bias in the current data.

### 3.2. Statistical Analysis

The Partial Least Squares (PLS-SEM) was used to test the hypotheses. The PLS-SEM is a comprehensive approach to multivariate statistical analysis that includes measurement and structural components to simultaneously examine the relationships between each of the variables in a conceptual model, which has the characteristic of multivariate analysis, that is, it involves some variables equal to or greater than three (J. Hair et al., 2010). In addition, the present study used the PLS-SEM because it facilitates theory-building (J. F. Hair et al., 2011). To perform the PLS-SEM analysis, WarpPLS (Version 8.0) was used; this software was used because, according to Kock (2014), it provides options to use different algorithms for the external and internal models in the calculation of the scores of the latent variables, such as the path coefficient and the parameters associated with the *p*-value, identifying and taking into account non-linear relationships in the structural model (Kock, 2011).

## 4. Results

The measurement and structural model evaluation were carried out through PLS-SEM to evaluate the quality of the reflective constructs, the convergent validity process, and the reliability of the construct were carried out that is, the internal consistency (W. W. Chin, 2010; J. F. Hair et al., 2011; Kock, 2015), considering that the authors establish minimum parameters in line with the literature. For example—for Loading (>0.7), the composite reliability (>0.7), Cronbach’s alpha (>0.7), the mean-variance extracted (>0.50), variance inflation factor (<5), and *p*-value (<0.05). Under these indicators, Table 2 shows compliance with all indicators. Regarding collinearity, the full collinearity VIF values ranged from 1.097 to 2.551, indicating values below the 3.3 threshold recommended for PLS-SEM models (Kock, 2014), thereby ensuring the absence of multicollinearity among the analyzed constructs and supporting the validity of the structural estimates. It should be noted that, in addition, when evaluating the quality of the measurement model using PLS-SEM, an adequate composite reliability was confirmed with a CR > 0.90 for the set of constructs, the AVE convergent validity >0.50, and an adequate discriminant validity for all scales, as shown in Table 2 and Table 3. This means the instruments worked properly for Peruvian university workers.

Discriminant validity indicates the extent to which each construct is distinct from other constructs in the model (W. W. Chin, 2010). To meet discriminant validity, the square root of the AVE for each construct must be greater than the highest correlation between the construct and other constructs in the model (W. W. Chin, 2010; J. F. Hair et al., 2011; Kock, 2014). Under these parameters, Table 3 is presented, which shows compliance with the criterion mentioned above; hence, the model is considered to meet the validity criterion.

Regarding the evaluation of the structural model, taking into account that it must be verified and report two preliminary criteria, the following is considered: the significance of the path coefficients and the R^2^ coefficient value for endogenous constructs. In the structural model, each hypothesis is associated with a causal link that represents the relationship between two constructs. Path coefficients and their corresponding *p*-values were calculated for each relationship in the model. Although the path coefficients must be significant, the R2 value is highly dependent on the research area. M. G. Chin (1998) suggests values of 0.67, 0.33, and 0.19 as substantial, moderate, and weak measures of R. In behavioral studies, a value of 0.2 for R2 is generally considered acceptable (J. F. Hair et al., 2014; Kock, 2013).

The present study’s R2 for the JPT, JPC, and JPP coefficients were 0.32, 0.28, and 0.07, respectively. Therefore, this R2 value is relatively moderate and acceptable. The results of this study suggest that the study variables (TL and IC) account for a high percentage of the variance in JPT, JPC, and JPP.

**Figure 2:**
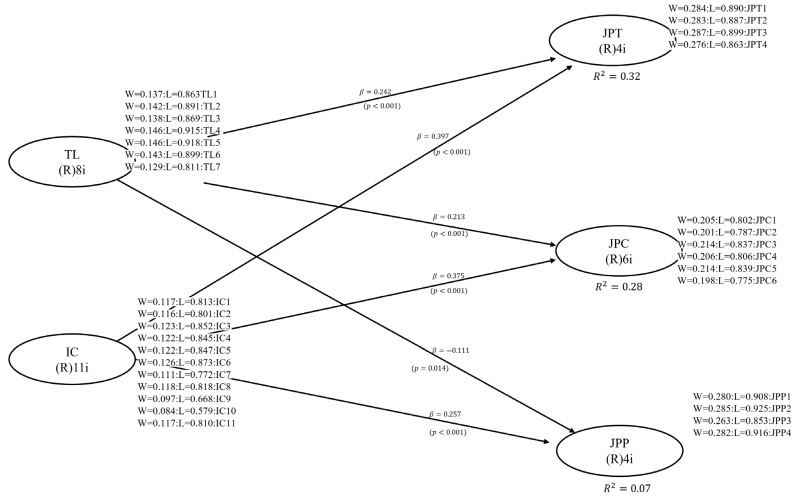
Structural model results.

Table 4 and Figure 2 show the results of the hypothesis tests and the evaluation of the path coefficients.

The findings provide evidence of the significant positive effects of TL on JPT (H1), TL on JPC (H2), IC on JPT (H4), IC on JPC (H5), and IC on JPP (H6). Furthermore, the same results show the negative and significant effect of TL on JPP (H3). The results indicate that transformational leaders who inspire and motivate their teams contribute to the efficient accomplishment of tasks (β = 0.242, H1) and encourage voluntary behaviors beyond the formal requirements of the role (β = 0.213, H2) among Peruvian university workers. In the same way, effective internal communication provides workers with the clarity and coordination necessary to fulfill their assigned tasks (β = 0.397, H4). It encourages proactive contributions and teamwork that extend beyond formal responsibilities (β = 0.375, H5). On the other hand, JPP has a positive effect with high scores that refer to a higher frequency of counterproductive behaviors; in this sense, the negative trajectory coefficient of TL towards JPP (β = −0.111, H3) shows a desirable organizational outcome, suggesting that higher levels of TL are associated with reduced counterproductive behaviors among university workers. In contrast to the positive effect of IC on JPP (β = 0.257, H6), which states that as internal communication improves, study subjects feel more able to recognize and report counterproductive situations, rather than indicating an actual increase in this behavior.

The six goodness-of-fit indices (Kock, 2014) have been used as global model fit indices at the 95% confidence level. In the case of the present study, the six fit indices suggested that the model fit was more than acceptable: Average Path Coefficient (APC) = 0.266, *p* < 0.001; Average R2 (ARS) = 0.223, *p* < 0.001; Average Adjusted R2-Squared (AARS) = 0.219, *p* < 0.001; Average Block Variance Inflation Factor (AVIF) = 1.299 (acceptable if ≤5, ideally ≤3.3); Average Full Collinearity Variance Inflation Factor (AFVIF) = 1.821 (acceptable if ≤5, ideally ≤3.3); and Tenenhaus GoF (GoF) = 0.404 (small ≥ 0.1, medium ≥ 0.25, large ≥ 0.36). A construct’s predictive validity can be confirmed when the associated coefficient R2 is greater than zero. This was the case for all values of the endogenous variables in the model, suggesting acceptable predictive validity.

## 5. Discussions

Based on the hypotheses that assume transformational leadership and internal communication predict job performance, the results section demonstrates this prediction. Thus, this article becomes further evidence that demonstrates the great importance that a leader assumes in various organizations; in this regard, Chi et al. (2023) and Choi et al. (2016) establish that transformational leadership generates improvements in workers that is, optimizes the attitudes that workers present in work environments, contributing this action not only to their well-being but also to the fulfillment of organizational objectives, since according to recent empirical evidence, this act becomes a way of emotional connection between the study variables (Rabiul et al., 2023). This research highlights the importance by demonstrating, with a theoretical basis, the role of the leader, whose function is translated into job performance and extends to the worker’s well-being.

This study also demonstrates that transformational leadership predicts job performance, a statement consistent with research by (Garad et al., 2022), who states that this style of leadership improves job performance, thus increasing efficiency in organizations, and that although transformational leadership had its beginnings at the end of the last century, it is believed that this is and continues to be the source of inspiration for employees, so that this variable can predict job performance (Burns, 2004; Zheng et al., 2013). In this context, it is stated that transformational leadership remains relevant and powerful, so it is important to use this leadership style to manage human resources. This is due to the need to navigate a competitive, global work environment, so cultivating a strong relationship between employees and employers is an essential factor in organizational success (López-Cabarcos et al., 2022).

The impact of transformational leadership on contextual performance has been demonstrated, characterized by workers’ initiative, enthusiasm, persistence, motivation, dedication, and proactivity. In this sense, the findings identified in this research are also supported by (Bolarinwa et al., 2013; Donkor et al., 2021; Al & Abdullah, 2020; Bastari et al., 2020) who indicate that the attachment generated as a result of the behavior of the transformational leader awakens in the workers a sense of belonging, which is why they seek to do a good job, putting their skills at the disposal of the organization due to the motivation they receive from the leader to generate a greater workforce in the right direction. In addition, Bhatti and Alyahya (2021) and Nazari et al. (2022) explain that when transformational leadership is present, workers become more deeply involved in their work, performing actions that go beyond fulfilling their responsibilities; that is, workers have a high availability to work hard; this means that the influence of this leadership style infuses energy and enthusiasm. In this way, the finding that establishes a prediction of transformational leadership in the decrease in counterproductive performance is also supported, thus assuming that the existence of a transformational leader allows for a reduction in counterproductive performance that is linked to actions such as off-task behavior, absenteeism, performing assigned tasks incorrectly, and other negative actions that undermine the morale and reputation of the organization (Koopmans et al., 2011; Z. Shao et al., 2012).

In addition, the role of this leadership style in task performance is evident; in this regard, the transformational leader can manage the appropriate orientation to new workers so that they can assertively fulfill the designated functions; in this way, workers could have over time the freedom to make decisions that allow them to optimize their work (Bisharat et al., 2016; Caniëls et al., 2018). Likewise, evidence has been found that supports this same idea by stating that transformational leadership influences performance, developing basic aspects that allow workers to fulfill their duties, with the responsibility they assume being a determinant for the success of the organization (Lee et al., 2018; Wang et al., 2017), thus creating a competitive advantage, and the workforce that a worker employs to fulfill his functions is a response to his commitment encouraged by an environment where there is a transformational leader (Manzoor et al., 2019; Othman, 2009). Furthermore, recent findings in the university setting confirm the direct link between transformational leadership and task performance; this means that when a leader promotes knowledge sharing, favorable conditions emerge that enable staff to go beyond their roles (Saif et al., 2024). Meanwhile, another study showed that leadership accounts for up to 49% of the variation in task performance among teachers, underscoring its importance in fostering emotional well-being (Yuntina et al., 2025).

From another perspective, it has been identified that internal communication predicts job performance; these results are also supported by (Obiunu & Yalaju, 2020; Wulandari, 2014), who state that communication is an act that must continue because it is observed that in all cases, it contributes substantially to performance, justifying this statement by finding that communication serves as a means to express the mission and vision of organizations, since this good practice allows solving problems and clarifying various situations related to work. To reinforce this idea, Ali and Zia-ur-Rehman (2014), Saraih et al. (2019) and Sotamba Romero (2025) argue that effective communication is a determinant that allows workers to fulfill the responsibilities of their job, so the role of this variable is crucial to connecting workers with the goals and objectives of the organization; that is, it is how workers make informed decisions, responding quickly to the major challenges that emerge at work. This provides evidence of the importance of internal communication as a useful component of job performance.

One notable finding is that internal communication shows a stronger predictive effect on task performance (β = 0.397) and contextual performance (β = 0.375) than does transformational leadership. The particular dynamics of university environments can explain this behavior. While leadership is demonstrated through motivational and inspiring mechanisms that shape attitudes and commitment among workers, internal communication serves as an immediate operational resource, enabling workers to understand their roles, coordinate tasks, and help fulfill the institution’s objectives. Specifically, within the university environment, where workers interact with various departments and hierarchies, clarity and transparency can have a greater impact on daily performance than leadership style. To support this interpretation, Welch and Jackson (2007) argue that communication connects performance, and that the leader’s strategies are consolidated into efficient, concrete results. This makes it clear that, in environments where workers have limited direct contact with their leader, such as universities, internal communication channels can partially replace or amplify leadership’s influence on performance, making it a more powerful predictor in these contexts. Another study examining the relationship between leadership and internal communication suggests that communication plays a particularly significant role in contexts with complex hierarchical structures, such as universities, providing evidence that communication is more than just a supporting factor; it becomes a powerful predictor of performance (Baihaqui & Fitrianingrum, 2025; Vicuña & Dominguez, 2025). Thus, it is clear that in an educational setting, communication improves employee performance.

Regarding explanatory power, the R^2^ values for counterproductive job performance were 0.07, a modest indicator. This suggests that although transformational leadership and internal communication are significant predictors of counterproductive behavior, they explain only a limited proportion of its variance. This fact is consistent with the multidimensional nature of counterproductive performance, which is additionally determined by individual factors such as personality traits and emotional regulation, as well as contextual factors such as organizational climate (Geraldo, 2022; Thibault & Kelloway, 2020). This same pattern is evident in the existing literature, which indicates that counterproductive behaviors are predicted by individual factors such as negative emotions, low emotional stability, and personality traits (Indrawiani et al., 2025; Spector, 2020), and other studies confirm that interpersonal conflicts and burnout lead to the same behaviors (Ghasemi & Herman, 2024). In this context, López Damián and Yee Villanueva (2024) note that every organization must address the external factors affecting employees to prevent negative behaviors from spreading to the workplace. In general, these previous findings suggest that variables such as leadership and communication provide only limited explanation for a construct as complex as counterproductive behaviors, which supports the low explanatory power identified in this study. This result highlights the importance of future studies that incorporate additional predictors to understand counterproductive behavior in university environments holistically.

## 6. Conclusions and Recommendations

Based on scientific literature that establishes that organizational success depends mainly on how the worker performs, this study analyzed two factors, transformational leadership and internal communication, which are considered predictors of job performance. The results indicate that the proposed model meets all relevant criteria for construct quality and reliability, and that its discriminant validity is acceptable. Furthermore, an important indicator of job performance was identified in the structural model, thereby rejecting the null hypotheses. The results for the prediction of Leadership show the significant positive effect of TL on JPT (Pat Coefficient 0.242), TL on JPC (Pat Coefficient 0.213), IC on JPT (Pat Coefficient 0.397), IC on JPC (Pat Coefficient 0.375), and IC on JPP (Pat Coefficient 0.375). Furthermore, the same results show LT’s negative and significant effect on JPP (Pat Coefficient −0.111).

From the results shown and since there is empirical evidence that supports that the TL predicts job performance, it is important to give the attribution that corresponds to the leader because, within his administrative task, he should not under any circumstances downplay the impact that his role has within the job performance of the workers, taking into account that the collective growth of an institution depends on them. Since it faces such a changing environment every day, this action is an adequate decision so that the entities can sustain themselves over time since it is advantageous for the organization to consider the positive influence of the TL on the contextual performance, recommending its adoption in the organizational model to obtain the best of the workers’ capacity so that they can adapt to the development of new work systems, facing it appropriately and placing their aptitudes in front of the adoption of the changes.

On the other hand, since the results indicate an inverse association between transformational leadership and counterproductive performance, it is important to maintain a balanced leadership style. This leadership, in addition to establishing internal communication policies, fosters a positive work environment, supports the development of workers’ creativity, and motivates them to adopt a positive attitude that contributes to institutional purposes.

Finally, taking into account that the qualities of a transformational leader have certain advantages, such as generating confidence in the worker to the point of making him an unconditional participant in each of the activities that involve the nature of the work, it is recommended that institutions can adopt a more proactive approach towards the identification of the transformational leader to enhance his skills, translating these actions into obtaining an effective and positive job performance that supports better management.

### 6.1. Theoretical Implications

Based on the findings, it is theoretically supported that the transformational leader inspires and motivates workers to reach high potential and remain committed to the objectives of their institution, empowering them and developing their skills, thus creating a positive work environment that fosters commitment and personal growth, which in turn improves job performance and contributes to the success of the organization. Additionally, effective internal communication is essential for good employee performance. It can measure clarity in expectations, align employees with organizational objectives, foster coordination and collaboration between work teams, and contribute to a positive work environment. On the other hand, poor internal communication can contribute to a deteriorating work environment and negatively affect employee performance. Meanwhile, effective internal communication increases employee motivation, job satisfaction, and commitment, translating into better individual and collective performance; in this context, organizations must practice clear and effective communication, promoting a culture of open communication and providing opportunities for participation and the exchange of ideas among workers, carrying out constant and constructive feedback practices, such as transparent communication of objectives and expectations, which are considered key elements for internal communication that promotes good job performance.

### 6.2. Practical Implications

The results have practical implications, specifically at the university’s administrative and human resources levels. First, given the positive effects of leadership on task and contextual performance, the university entity should invest in leadership development programs for its administrative staff, emphasizing inspiration, individualized consideration, and intellectual stimulation. This practical action involves structured mentoring programs, leadership coaching, and 360° evaluations that provide assertive feedback to identify areas for improvement.

Secondly, after identifying the predictive effect of internal communication on task and contextual performance, university entities should implement a clear, two-way communication protocol and schedule computer meetings to ensure that any institutional objective can be communicated transparently to all staff. In addition, they should incorporate digital communication platforms that facilitate real-time information exchange.

Third, given the negative effect of transformational leadership on counterproductive performance—which suggests that when there is solid transformational leadership, undesirable behaviors are reduced—university entities should periodically train their leaders to focus on building trust and a sense of purpose among workers.

Finally, the positive effect of internal communication on counterproductive performance should be interpreted with caution: it suggests that in environments with open and effective communication channels, workers feel more empowered to recognize and report counterproductive behaviors. In this sense, the recommendation is that university managers establish formal and safe internal reporting mechanisms, which represents not a real increase in negative behaviors, but an institutional opportunity for continuous improvement and transparent management.

### 6.3. Limitations and Future Research

Although this research examines transformational leadership and internal communication as factors predicting job performance, the workers’ direct perspectives on these variables are unknown, so an accurate picture of their perceptions of their leader and the communication style in their occupational area could not be obtained. This gap offers an opportunity for new research that examines these workers’ perspectives in depth through a qualitative study. Furthermore, no comparative analysis has been conducted to examine workers’ opinions about their occupational areas.

On the other hand, this research analyzed job performance, a variable much analyzed with job satisfaction and commitment; however, the latter was not included because this research opted for a parsimonious model focused on identifying its direct effect on job performance from transformational leadership and internal communication, so future research could analyze the mediating mechanisms that drive job performance.

Finally, the results show a modest R^2^ value for counterproductive job performance (JPP = 0.07), which means that transformational leadership and internal communication explain only a small proportion of the variance in this dimension. This is likely due to the multidimensional nature of counterproductive performance, which is influenced by additional individual and contextual factors not included in the proposed model, such as personality traits, organizational climate, and job satisfaction. Therefore, future research should incorporate these variables to identify a more complete explanation of counterproductive behavior in university settings.

## Figures and Tables

**Table 1:** Sociodemographic characteristics of the study population.

		Frequency	Percentage
Sex	Female	168	43.60
	Male	217	56.40
Total		385	100.0
Status	Married	232	60.3
	Single	133	34.5
	Divorced	17	4.4
	Widowed	1	0.3
	Co-habitant	2	0.5
Total		385	100.0
Age	19–23	11	2.9
	24–28	57	14.9
	29–33	51	13.2
	34–38	52	13.2
	39–43	64	16.6
	44–48	58	15.1
	49–53	45	11.8
	54–58	22	14.6
	59–64	20	5.2
	65–68	5	1.4
Total		385	100
Occupational Area	Professor	152	39.5
	Administrative support staff	169	43.9
	Service area support staff	64	16.6
Total		385	100

**Table 2:** Measurement model evaluation results.

Item	Loading	*p*-Value	CR	Cronbach’s	AVE	Full Collinearity VIFs
TL1	0.863	<0.001	0.967	0.961	0.785	1.414
TL2	0.891	<0.001				
TL3	0.869	<0.001				
TL4	0.915	<0.001				
TL5	0.918	<0.001				
TL6	0.899	<0.001				
TL7	0.811	<0.001				
IC1	0.813	<0.001	0.949	0.940	0.630	1.717
IC2	0.801	<0.001				
IC3	0.852	<0.001				
IC4	0.845	<0.001				
IC5	0.847	<0.001				
IC6	0.873	<0.001				
IC7	0.772	<0.001				
IC8	0.818	<0.001				
IC9	0.668	<0.001				
IC10	0.579	<0.001				
IC11	0.810	<0.001				
JPT1	0.890	<0.001	0.935	0.907	0.783	2.551
JPT2	0.887	<0.001				
JPT3	0.899	<0.001				
JPT4	0.863	<0.001				
JPC1	0.802	<0.001	0.918	0.893	0.653	2.327
JPC2	0.787	<0.001				
JPC3	0.837	<0.001				
JPC4	0.806	<0.001				
JPC5	0.839	<0.001				
JPC6	0.775	<0.001				
JPP1	0.908	<0.001	0.945	0.922	0.811	1.097
JPP2	0.925	<0.001				
JPP3	0.853	<0.001				
JPP4	0.916	<0.001				

**Table 3:** Discriminant validity.

	LT	IC	JPT	JPC	JPP
LT	0.886				
IC	0.497	0.793			
JPT	0.404	0.515	0.885		
JPC	0.345	0.475	0.744	0.808	
JPP	−0.066	0.17	−0.056	0.052	0.901

**Table 4:** Hypothesis testing results.

	Hypothesis	Pat Coefficient	*p*-Value	Decision
H1	TL-JPT	0.242	<0.001	Accepted
H2	TL-JPC	0.213	<0.001	Accepted
H3	TL-JPP	−0.111	0.014	Accepted
H4	IC-JPT	0.397	<0.001	Accepted
H5	IC-JPC	0.375	<0.001	Accepted
H6	IC-JPP	0.257	<0.001	Accepted

## Data Availability

Data are available on request from the authors.
